# The epidemic of HIV/AIDS in developing countries; the current scenario in Pakistan

**DOI:** 10.1186/1743-422X-8-401

**Published:** 2011-08-12

**Authors:** Muhammad Z Yousaf, Sadia Zia, Masroor E Babar, Usman A Ashfaq

**Affiliations:** 1 Institute of Biochemistry and Biotechnology University of Veterinary and Animal Sciences, Lahore; 2 Division of Molecular Medicine, National Centre of Excellence in Molecular Biology, University of the Punjab, Lahore, Pakistan

## Abstract

HIV (Human Immunodeficiency virus) causes (acquired immunodeficiency syndrome) AIDS, in which the immune system of body totally fails to develop any defense against the foreign invaders. Infection with HIV occurs by transfer of blood, semen, and breast milk. HIV/AIDS is a global problem and it results nearly 25 million deaths worldwide. Developing countries like Pakistan have issues regarding Public Health. Currently, epidemic of HIV/AIDS is established in Pakistan and there is a threat of an expanded HIV/AIDS outbreak in the country. The major reason is that population is engaging in high-risk practices, low awareness about HIV/AIDS, and treacherous blood transfusion practices. A supplementary threat to Pakistan is India because both sharing a border and India is facing a rapidly growing HIV/AIDS epidemic. Local NGOs, National and International organizations are warning that in near future Pakistan may experiences bad situation regarding HIV/AIDS.

In the present article we focused current situation of surveillance of HIV/AIDS, its virology, genotype, diagnostics, high-risk groups, reasons of vulnerability in Pakistani population, and the role of different national and international organizations in this situation.

## Background

HIV belongs to *Lentivirus*, which are also known as "slow virus". The name indicates there mode of action as they enters into body and remain in it for longer period of time. They have unique property of being inserting the information into the DNA of host cell and also have the ability to replicate in non-dividing cells. Due to these characteristics they are considered to be the most efficient gene delivery vector [1]. HIV infects defense/immune system cells such as CD4+ T cells, macrophages and dendritic cells [2]. The CD4+ cells play a crucial role in the maintenance of immune system. After infection, HIV uses CD4+ cells as host to make copies and infect other cells. This leads to the reduction of CD4+ cells in body and immune system totally collapse [3]. The development from HIV to AIDS is checked by the rapid decline of CD4+ cells [3].

### Types of HIV

Two types of HIV has been characterized; HIV-1 and HIV-2. HIV-1 is the most virulent and pathogenic strain. Worldwide, the predominant virus is HIV-1, and generally when people refer to HIV without specifying the type of virus they will be referring to HIV-1. The relatively uncommon HIV-2 type is concentrated in West Africa and is rarely found elsewhere. The reason behind is that HIV-2 weaken the immune system slowly than HIV-1 [4]. The HIV-1 is further divided into 4-groups; a) major group M, b) Outlier group O, c) Group N, d) Group P. These groups have been identified in there envelop region. Group M is further divided in to A, B, C, D, F, G, H, J and K. in Asian countries B and C are the predominant clades of HIV-1. But in Pakistan HIV-1 is dominant in Pakistan as compare to other clades, this was found more in IDUs in Karachi [5]. HIV-2 has also 8 clades from A to H, out of these clades only A and B are epidemic [6].

### HIV Virology and Life Cycle

**Figure 1:**
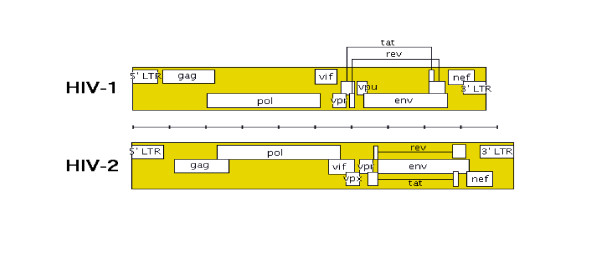
**The diagram is based on excellent mapsof HIV-1, HIV-2 and SIV genome available at http://www.hiv.lanl.gov/content/immunology/pdf/2000/intro/GenomeMaps.pdf**.

The identification of HIV led the concentrated activity in the field of molecular virology. HIV is different in structure from other retroviruses. This is roughly spherical with a diameter of about 120 nm [7,8]. It contains three (3) structural and six (6) genes which encodes the at least fifteen (15) viral proteins and control the ability of HIV to infect the cell [9]. HIV is composed of two copies of positive single stranded RNA (Figure 1). The RNA is tightly bounded with nucleocapsid proteins and the essential enzymes for the development of virion such as; reverse transcriptase, proteases, ribonucleases and integrase [10].

**Figure 2:**
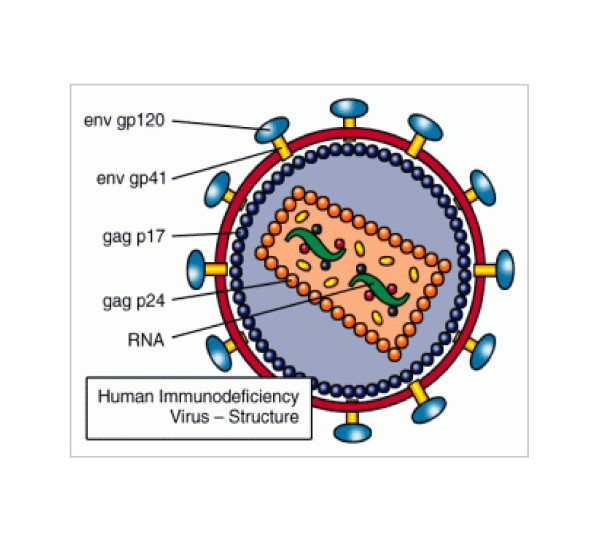
**The diagram is based on structural glycoproteins of HIV available at http://www.avert.org/hiv-virus.htm**.

The two RNAs are surrounded by the viral envelope, which is composed of phospholipids. Envelop contains embedded protein from the host cell and about 70 copies of complex HIV proteins. These proteins are called as envelop proteins or *env *[10]. The RNA genome consists of seven (7) genomic structural elements and nine (9) genes [11]. These are seven (7) in number including: LTR, TAR, RRE, PE, SLIP, CRS and INS. These are nine in number including; *gag, pol, env, tat, rev, nef, vif, vpr, vpu and tev *[11]. gag is a group specific antigen which encodes gag polyprotein. Tat is Transactivator of HIV gene expression. The env protein consists of cap made up of three molecules called glycoprotein (gp) 120, and a stem consists of gp 41 molecules that enables the virus to attach and fuse with target cells [11]. Outside the human cells, the HIV exists as roughly spherical particle. HIV particles surround them selves with a fatty material known as envelop. Nearly 72 little spikes projecting out from envelop which are formed by the gp120 and gp 41 protein (Figure 2). Below envelop there is a layer of matrix made up of protein P17. The viral capsid is usually of bullet shaped and made from the protein P24. Inside the core there are three enzymes reverse transcriptase, integrase and protease which are require for HIV replication [12].

HIV attached its life cycle by attaching the CD receptor, fuse within the cell and release its RNA into host cell. The enzyme transcriptase converts the RNA into DNA. The newly formed DNA enters the host cell nucleus and enzyme integrase hides it within the host cell's DNA. This integrated HIV DNA is called provirus, and this provirus may remain inside the body for several of years. When the provirus receives the signals to be active, they use host cell RNA polymerase to make messenger RNA [13]. The mRNA makes long chains of HIV proteins which cut with the help of proteases. The newly assembled virus pouches out from the host cell [13].

### Situation of Pakistan regarding HIV/AIDS

The total population of Pakistan is 168.79 million at the end of 2009 with an average annual growth rate of 1.9% [13]. In the ranking of most populated nations; Pakistan stands at 6^th ^position in the world. Pakistan is divided into four provinces viz., Punjab, Sindh, Khyber Pkahtunkhwa and Balochistan; two independent states of Azad Jammu Kashmir and Gilgit-Baltistan; and federal territories of Federal Administrated Tribal Area [FATA] and Islamabad [14]. The most populated Provinces of Pakistan are Punjab and Sindh with high HIV/AIDS prevalence. The mortality rate is 64 per 100,000 live births with life expectancy at birth of 66 years [15]. The literacy rate in Pakistan is 54% with 0.562 human development indexes and 0.537 gender development index [16].

Pakistan is at high risk for an HIV/AIDS epidemic due to the presence of several socioeconomic conditions conducive to the spread of HIV, including poverty, low level of education and high unemployment [17]. The first case of HIV/AIDS was reported in 1987 in Pakistan. The National AIDS control Program of Government of Pakistan reports a cumulative total of 1813 HIV/AIDS cases and an estimated HIV prevalence of 0.1% as of 2001 [18]. A recent study of USAID estimated the total population of Pakistan in the mid of 2010 was 177 million with estimated population living with HIV/AIDS is 96000 at the end of 2007 [17,19], However, the reliability of available data is limited, and actual rates of HIV/AIDS infection in Pakistan is much higher than official report suggested. The main reason is that; small number of people is registered with NGOs having HIV/AIDS. Most of them do not register them selves due to the threat of social isolation, and many of them do not aware that they are living with this disease.

### The Epidemic Groups

There are 11 major core groups which are considered as high risk factors for HIV/AIDS transmission. The IDUs (intravenous injection drug users) and MSWs (male sex workers) are the main groups for HIV/AIDS in Pakistan [19,20].

### a) IDUs and their Spouse

In 2008 it was estimated that there are about 100,000 streets based IDUs in Pakistan of which 21%have HIV infection [21]. According to another report the IDUs embody the core group driving the epidemic and the highest prevalence of 20.8% [22].

About 95% of IDUs are sexually active in Pakistan and 38% have regular sexual partner [21]. The IDU's spouse are at moderate risk of acquiring HIV from their husbands via infrequent but unprotected sexual intercourse, the low condoms use and lack of HIV protection knowledge [23]. The IDUs usually go to local doctors or quakes for injections. They use the cocktail of Diazepam and Phenirimine which is readily available in Pakistani pharmacies that some times sell it without prescription [23]. The most spouse of IDUs also use drugs along with their husbands using same injections. Most of spouses were aware that they are drug users but they used to control the actual physical pain, and with the passage of time they got addicted to it.

### b) Male Sex workers (MSWs)

Sex work is illegal in Islamic Republic of Pakistan, but unfortunately, it has a long history and is booming industry in the country. The most alarming thing is that, there is high rate of male to male sex in Pakistan as compare to male to female. The MSWs are more in big cities of Pakistan like Karachi, Lahore and Hyderabad. According to a survey conducted in 2007 in Rawalpindi and Abbotabad, there are 917 MSWs in these cities [24]. The MSWs are further divided in to further groups;

#### Hijras (also known as Khusras)

Hijras is the collective term used in Pakistan for men who are transgender, eunuch, transvestites, hermaphrodites or intersexes. Hijras are biological males who are often fully castrated (eunuch) [25]. They are respective partners in anal sex and high risk factor of HIV transmission.

#### Zenanas and Chavas (Khotkis)

these are the men but recognize themselves with the female gender. They depict themselves as women and commercially sell sex with multiple partners. They often married to women and have children. In sex work they assume the female gender role [26]. Chavas can switch their sexual roles.

#### Giryas (banthas)

these are the people who marry with Hijras and Zenanas and assume the role of husband.

#### Maalishias

the young and mature males work as massagers and also sell sex. Male sex workers are emerging as a second highest risk group for HIV infection after IDUs [27].

### c) Female Sex Workers (FSWs)

Extrapolation of surveillance data in 2005 suggests that there are around 136,300 FSWs nationwide [28]. A study of FSWs in 8 major cities of Pakistan estimated that there were about 34,500 FSWs in these cities. These are 760 in Quetta and 14,150 in Lahore [29]. The high number of FSWs are reducing in traditional brothels and setting in residential areas and streets. Where as hotels, parlors, roadsides, chowks, chowk crossings, markets, roadsides, parks, railway stations, bus stands, mazaars, hospitals, parking are also emerging as a major assess areas for FSWs and clients in major cities [30]. The easy access to these FSWs is due to ban on *Kothikhanas *and dancing. They are residing among the civil society and commercial areas. These FSWs are between the ages of 13-45 years.

### d) Migrant

Migrant are those who are low skilled rural men and travel to other countries for work. They remain away from families for years and this lead them to indulge in such sexual activities. Due to unsafe sexual intercourse and they become victims of HIV/AIDS. Most of migrants are from remote areas of Punjab and tribal areas of FATA, Balochistan and Khyber Pkahtunkhwa. All the patients, who are registered in Pakistan, are those migrants who are departed back after mandatory HIV testing in abroad. The major proportion of Pakistani migrants resides in Gulf countries. Nearly 2 million people are working in Middle East [31]. These migrants also transmit the HIV/AIDS to their spouse.

### e) Truck Drivers

The huge population of tribal areas of Pakistan is truck drivers. They travel faraway from their houses even go to different countries adjacent to Pakistan. These truck drivers are at the high risk of acquiring and transmitting the HIV/AIDS from unprotected sex with workers and casual partners during their prolonged absence from home [31]. The sex partners of truck drivers are young boys, worked as helpers. Some times they also involve in sex with females and other male sex workers. They debut sex around 17 years, 60% are married and quarter engage in commercial and non-commercial extra martial sex and rarely use condoms [31]. The same study found 1% HIV prevalence among truck drivers in Lahore [32].

### f) Miners

According to Balochistan AIDS control program there are more than 100,000 miners nationwide. They work away from their home for months. An assessment from one location found that 42% had sex with their colleagues [33].

### g) Prisoners

In the year 2009 Government of Sindh has established a program for delivering HIV/AIDS prevention services to jail inmates at Karachi, Hyderabad and Sukkur. In this program inmates are informed of the threat of HIV infection and methods of prevention. They are provided access to confidential volunteer counseling and testing services for HIV/AIDS to help them know of their current HIV status. They test the prisoners from the age of 10 to 59 and above. Out of 4987 prisoners 49 were HIV/AIDS positive and most of them were IDUs [34].

### h) Unsafe and Invasive medical Practices

According to World Bank report of June, 2005 Pakistan has a high rate of medical injections - around 4.5 per capita per year. Studies indicate that 94 percent of injections are administered with used injection equipment. Use of unsterilized needles at medical facilities is also widespread. According to WHO estimates, unsafe injections account for 62 percent of Hepatitis B, 84% of Hepatitis C, and 3 percent of new HIV cases [35]. The most important thing is that; in Pakistan, the roadside dentists pose HIV threat to the people. Government of Pakistan, in 2006, figured out that there are 6,761 dentists in the country for the population of 155 million. This indicates that 1 dentist for 23,000 patients [36]. The main reasons of approaching people to these roadside unqualified doctors are; the unavailability of qualified dentists and their fee charges. The roadside dentists do not take care of hygienic issues and they use the equipments to many patients. This practice is more dangerous for spreading HIV/AIDS infection.

### i) Barber Shops, Beauty Saloons

Barber is a person whose occupation is to cut any type of hair, give shaves, and trim beard. A barber differs from a hairdresser whose business is generally restricted to cutting and styling hair. Barbering is an ancient profession. The earliest records of barbers show that they were the foremost men of their tribe. Specific HIV-risks of barbering procedures relating to HIV transmission has been documented in Nigeria and other African and Asian countries [37]. The main reason for this is reuse of razors, blades and lack of awareness among barbers about HIV transmission. A recent study was conducted in 250 beauty saloons of Karachi and found that the greater risks of cuts and snips took place during manicure and pedicure. These expose areas are the greater risks for getting HCV and even HIV [38].

### j) The Transmission of HIV from Mother to Infant

According to a estimation, there are 2.3 million children living with HIV/AIDS in the world today. The vast majority of these children are living in Asia and southern Africa. Mother to child transmission during pregnancy, child-birth, or breast-feeding is the most important source of infection in children. HIV/AIDS has not spared Pakistan, and an increasing number of women and children infected with HIV are being reported from around the country. Although the documented number of parentally acquired cases among children in Pakistan is only 40, several factors contribute to suggest that this number is a significant under-estimate. These include the lack of awareness about HIV/AIDS among the general population and among health care professionals, the stigma associated with HIV/AIDS, lack of diagnostic testing facilities, and the difficulties of making a diagnosis in children, especially in a country where malnutrition rates are as high as 30% among children under 5 years of age [39].

### k) Prevalence of HIV in Pakistani Youth

Pakistani youth just like other young people all over the world, are also curious about sex and drugs. During adolescent age of formation of values and habits, they are heavily influenced by their peers. Another study of National AIDS control program in 2005 suggested that only in Karachi streets children debut sex around the age of 13-15 years and 30% had sold sex to men and women [40]. About 80% of them do not use condom and if they used it the decision was made by elder partner [40]. The reasons behind this bitter reality are unemployment, easy availability of narcotic drugs, economic frustration that influence the young people to engage in unsafe behavior which may put them at increased risk of HIV infection [41].

### Diagnosis Methods of HIV/AIDS in Pakistan

NACP (National AIDS Control Program) has been involved in gigantic task of limiting the spread of HIV/AIDS in Pakistan [42]. In Pakistan the main purpose of HIV testing is to guide the NGOs, Private and Government Hospitals and the other people that how to deal with HIV/AIDS patients [43]. There are different methods to diagnose HIV/AIDS in different research institutes and hospitals in Pakistan. The most commonly used methods include molecular and serological detections. The standard of testing people with different ages which is practiced in Pakistan is as followed;

### Less than 18 months of age

The newborns of HIV infected mothers are more suspected to have this infection. These newborns are tested from 6 weeks after birth through PCR. Antibody tests can be used from 12 months for screening out the HIV negative children but the results are not supposed to be accurate because at this age most infants lost the maternal antibodies and the positive result on ELISA test usually indicate HIV infection [43].

### 18 months of age up to 12 years

At this stage usually ELISA or Western Blot is used to detect the HIV/AIDS. Those children who are more than 18 months and still being breastfed should cease breast feeding abruptly before testing with antibody test. But this test should be repeated after 6 weeks of cessation of breast feeding [43].

### Counseling of Children under 12 years of age

The counseling process is very much important before and after the test of children. If the children are not mature enough they are counseled in the presence of parents.

### Diagnostics Centers for HIV/AIDS in Pakistan

The two most common techniques for HIV/AIDS testing are available at different institutes of Pakistan under the Governance of National AIDS Control Program Pakistan.

The facility is available at Pakistan Institute of Medical Sciences (PIMS) Islamabad and Armed Forces Institute of Pathology (AFIP) Islamabad, Shaukat Khanam Memorial Hospital Lahore, Sindh Institute of Urology & Transplants (SUIT) and Agha Khan Hospital Karachi.

The facility of ELISA is available at every tertiary care hospital including; Service Hospital, Mayo Hospital and Shaukat Khanam Hospital of Lahore. HIV Treatment Center DHQ, Sargodha, Sindh AIDS Control Program; Indus Hospital; Agha Khan Hospital of Karachi, Bolan Medical Complex, Quetta and Hayatabad Medical Complex, Peshawar [43].

### The Reasons of High Vulnerability of HIV/AIDS in Pakistan

Although, HIV/AIDS prevalence appears to be low in Pakistan but there is need to establish some more accurate action plans for this. In Pakistan, the social structure and conditions include widespread poverty, significant power imbalance between men and women, low level of education, and challenges in the areas of government and human rights [44].

**a) Poverty **is a major development concern in Pakistan, and this is also a major facilitating factor in spread of HIV. Recent documentation suggests that poverty is increasing in Pakistan, about 36 million people living below the poverty line. The poor suffer not just limitations in income; they also lack basic facilities and amenities which allow for a full and meaningful existence.

**b) Gender Inequalities **may also play a significant role in the further spread of HIV/AIDS in Pakistan. Pakistani women in general have lower socioeconomic status, less mobility and less decision-making power than Pakistani men, all of which contributes to their vulnerability to HIV. Because of gender disparities in educational enrollment, the 35% of women are literate as compared to 59% of men in Pakistan.

**c) Other Reasons **against this backdrop of poverty, gender inequalities, and low literacy rate. The general public in Pakistan is vulnerable to HIV/AIDS due to several other common behavioral patterns and risk situations. For example, rarity of condom usage, unhygienic conditions and unsafe medical practices.

### Suggestions and Recommendation

While the current HIV levels are low in Pakistan but there is need to act to much avert a much larger epidemic in future. Based on all studies and findings and National and International reports by NGOs we recommend some important facts;

#### a) General Awareness and Behavioral Change

There is need to change the behavior of people in there daily life routine. People should be educated through radio, TV, newspapers, seminars and health related workshops about the importance of hygiene in there lives. We should facilitate the people through proper channels that they are important and there lives are more valuable like any other healthy person in Pakistan. There is need to target the vulnerable groups for there proper counseling.

#### b) Ensure the Safe Public Health Care Practices

This is true as a developing country Pakistan is facing immense problems relating to public health. The poor hygiene conditions in hospitals, easy availability of drugs without any prescription from pharmacies all these are the hot issues. But still to fight against HIV/AIDS these areas should be critically checked by Government agencies and non-governmental organizations. The rate of "Atties"are also very high in rural areas of Pakistan, who do not use any sterilized equipments during there treatment. These should be ban so that the risk factor can be reduced.

#### c) Sexual Health Care Centers

Like other developed countries there should be sexual health care centers and rehabilitation centers in Pakistan. These centers should work along the governmental and non-government organizations in red areas of Pakistan where rate of HIV/AIDS victims are more. These centers should register those victims and provide them not only physical treatment but also mental treatment. The big hurdle in Pakistan is that people are not ready to register themselves as HIV/AIDS victims because of social pressure and died. These centers should aware the people about the safe sex practices with their partners and use of condoms.

#### d) Say "No" to stigmatization barrier

People here in Pakistan are drive by there social status. The people who are living below the poverty line are more stressed, frustrated and more conscious about there livelihood. If these people involved in such activities than, they face the social stigma and remain hidden. There is need to educate the people that HIV/AIDS may not be treatable but if they are suffering with it, they are not thought to be isolated from rest of the society. Because there stigma can cause more problems for other people and can increase the risk of prevalence of HIV/AIDS in Pakistan.

#### e) HIV study centers

The Government of Pakistan should take initiative along with NGOs in the establishment of HIV study centers. These centers should be design district wise. The centers should be assigned with the tasks to register the HIV victims in their respective districts. These centers must collaborate with research institutes so that, the constructive work should embark on with reference to Pakistan.

## Conclusion

This is the common thought in the minds of Pakistani people that as an Islamic Republic, Pakistan is protected from HIV/AIDS. This is true that Islam is against pre-marital sex or extra-marital sex and also homosexuality, and this is a valuable barrier against HIV/AIDS. But still there is a threat of prevalence of this disease in Pakistan.

The HIV/AIDS counseling and testing is the best way to prevent this disease in Pakistan. There should be availability and accessibility of antiviral treatment so that people suffering with HIV can enjoy better life. The government of Pakistan should play there role because the successful and comprehensive HIV prevention program needs political leadership as this will be very much helpful is political personalities talk about HIV/AIDS on public places. This is eleventh hour to take measures against this disease to save future of Pakistan.

## Abbreviations

**HIV**: Human Immunodeficieny Virus; **AIDS: **Acquired Immunodeficiency Syndrome.

## Competing interests

The authors declare that they have no competing interests.

## Authors' contributions

MZY conceived the study and drafted the manuscript. SZ searched the literature and helped in manuscript write-up. MEB and UAA critically review the manuscript. All authors read and approved the final manuscript.

## Author's Information

Muhammad Z Yousaf (PhD Molecular Biology), Masroor E Babar (Director IBBT, UVAS), Usman A Ashfaq (PhD Molecular Biology), Sadia Zia (M-Phil Scholar)
